# Anlotinib for refractory advanced non-small-cell lung cancer: A systematic review and meta-analysis

**DOI:** 10.1371/journal.pone.0242982

**Published:** 2020-11-30

**Authors:** Guocan Yu, Yanqin Shen, Xudong Xu, Fangming Zhong

**Affiliations:** 1 Department of thoracic surgery, Zhejiang Chinese Medicine and Western Medicine Integrated Hospital, Hangzhou, Zhejiang, China; 2 Department of tuberculosis, Zhejiang Chinese Medicine and Western Medicine Integrated Hospital, Hangzhou, Zhejiang, China; Mansoura University, EGYPT

## Abstract

**Objective:**

To assess the efficacy and toxicity of anlotinib for the treatment of refractory advanced non-small-cell lung cancer (NSCLC).

**Methods:**

We systematically searched databases for randomized controlled trials on anlotinib treatment for patients with advanced NSCLC published until November 6, 2020. Articles were assessed and data were extracted independently by two investigators. Further, we analyzed hazard ratios (HRs) for progression-free and overall survival (PFS and OS, respectively). In addition, we analyzed risk ratio (RR) for overall response and disease control rates (ORR and DCR, respectively) and the odds ratio (OR) for the main adverse events (AEs) using RevMan 5.3 software.

**Results:**

This analysis included 594 patients from three clinical studies. The pooled HRs for PFS and OS were 0.27 (95% confidence interval (CI): 0.22–0.33, P < 0.001) and 0.68 (95% CI: 0.56–0.83, P < 0.001), respectively, indicating that anlotinib administration significantly improved PFS and OS in patients with advanced NSCLC. The pooled RRs for ORR and DCR were 11.62 (95% CI: 2.75–49.14, P < 0.001) and 2.30 (95% CI: 1.91–2.77, P < 0.001), respectively, indicating that anlotinib administration in patients with advanced NSCLC improved ORR and DCR. The pooled OR for AEs of grade 3 or higher was 2.94 (95% CI: 1.99–4.35, P < 0.001), indicating that AEs of grade 3 or higher were more prevalent in the anlotinib group than in the placebo group.

**Conclusion:**

Anlotinib, an effective choice of third- or later line therapy for patients with refractory advanced NSCLC, provides clinical benefits in terms of PFS, OS, ORR, and DCR. AEs associated with anlotinib were tolerable.

## Introduction

Lung cancer is the most common cancer and the leading cause of cancer-related death worldwide [1]. In 2018, more than 2 million new cases of lung cancer were reported worldwide, and the number of deaths due to lung cancer reached 1.76 million [1]. Non-small-cell-lung cancer (NSCLC) accounts for approximately 80%–85% of all cases of lung cancer, with the majority of cases presenting as locally advanced or metastatic NSCLC at the time of diagnosis [2]. Surgery can be successful for a minority of these patients, but the 5-year survival has been reported to be under 23% for patients with advanced NSCLC who receive surgical treatment [3].

Platinum-based dual-drug chemotherapy is the traditional standard approach for the treatment of advanced NSCLC; however, the corresponding 5-year survival rate remains very low at <5% [4]. With the development of precision medicine, targeted therapy, and immunotherapy, treatment strategies for advanced NSCLC have evolved. As per the patient’s constitution, mutation status of the driver genes (such as epidermal growth factor receptor (EGFR)), and the expression level of programmed cell death-ligand 1 (PD-L1), the first- and second-line treatments include targeted treatment, dual-drug chemotherapy with platinum-containing compounds, or immunotherapy [5–7]. However, EGFR mutations are reportedly only present in 10%–30% of patients with NSCLC [8]. Furthermore, immunotherapy is very expensive; thus, the benefits of targeted therapy and immunotherapy are minimal for patients who are negative for driver gene mutation and have a low income. There is no standard treatment regimen for patients who do not respond to second- or later line treatment [9], although many receive chemotherapy in practice [10].

In the past few decades, increasing evidence has indicated the role of neovascularization in the growth, proliferation, and metastasis of various solid tumors [11]. Antiangiogenic drugs can act on the tumor microenvironment, degrade existing tumor blood vessels, and inhibit tumor neovascularization [12]. Vascular endothelial growth factor (VEGF) and VEGF receptor (VEGFR) are important targets of cancer therapy, and inhibition of VEGF and VEGFR has been proven to be effective against advanced NSCLC in several studies [13,14].

Anlotinib is a novel, small-molecule oral multitarget tyrosine kinase inhibitor (TKI) of VEGFRs 1–3, fibroblast growth factor receptors 1–4, platelet-derived growth factor receptors α and β (PDGFRs α and β), and stem cell-factor receptor (c-Kit). Anlotinib could inhibit tumor angiogenesis and proliferation [15,16] to prolong the survival time of patients by inhibiting tumor growth through restriction of tumor blood supply. In addition, anlotinib has been shown to be efficient and safe for multiple tumor types, such as ovarian, cervical, and endometrial cancers [17], and its use as a third- or later line treatment has been reported for patients with refractory advanced NSCLC [18]. This meta-analysis aimed to assess the efficacy and toxicity of anlotinib for refractory advanced NSCLC.

## Materials and methods

### Design

We conducted a systematic review and meta-analysis to assess the efficacy and toxicity of anlotinib for refractory advanced NSCLC. This study was conducted following the Preferred Reporting Items for Systematic Reviews and Meta-Analyses (PRISMA) statements [19]. All materials for this study were derived from the published literature; therefore, ethical approval was not required.

### Information sources

We searched for potential eligible studies in China National Knowledge Infrastructure (CNKI), Wanfang Database, Cochrane Library, Embase, and PubMed up to November 6, 2020. Additionally, the references in the relevant reviews were assessed to identify other potential studies.

### Search strategy

Guocan Yu and Yanqin Shen conducted comprehensive searches independently. There were no language or date restrictions in the search process. The search strategy for PubMed is described as follows:

#1 “Carcinoma, Non-Small-Cell Lung” [Mesh]
#2 “Carcinoma, Non Small Cell Lung” OR “Carcinomas, Non-Small-Cell Lung” OR “Lung Carcinoma, Non-Small-Cell” OR “Lung Carcinomas, Non-Small-Cell” OR “Non-Small-Cell Lung Carcinomas” OR “Nonsmall Cell Lung Cancer” OR “Non-Small-Cell Lung Carcinoma” OR “Non Small Cell Lung Carcinoma” OR “Carcinoma, Non-Small Cell Lung” OR “Non-Small Cell Lung Cancer”
#3 #1 OR #2
#4 “anlotinib” [Supplementary Concept] OR AL3818
#5 #3 AND#4

The CNKI, Wanfang database, Cochrane Library, and Embase used similar search formulae.

### Eligibility criteria

#### Type of study

This study primarily included prospective clinical studies, such as clinical randomized controlled trials (RCTs) that had sufficient data available for extraction. Thus, retrospective studies, case reports, articles published in non-Chinese or non-English languages, conference reports, non-RCTs, abstracts without full texts, and studies that did not report a main outcome were excluded.

#### Participants

Patients with pathological confirmation of refractory advanced NSCLC who failed first- or second-line treatment were included. There were no restrictions on sex, age, and ethnicity.

#### Interventions

Treatment containing anlotinib served as the intervention in the observation group. Placebo or other treatments that did not contain anlotinib served as the interventions in the control groups.

#### Outcomes

The main outcomes included the median progression-free survival (PFS) and overall survival (OS), and the secondary outcomes included the objective response rate (ORR), disease control rate (DCR), and adverse events (AEs).

#### Study selection

ENDNOTE X9.2 literature management software was used to manage the relevant candidate articles from the search. Two investigators (Guocan Yu and Yanqin Shen) independently assessed candidate articles through review of titles and abstracts, followed by the full text according to the eligibility criteria. Any disagreements between the two investigators were resolved by discussion with a third investigator (Fangming Zhong).

#### Data extraction

The same two investigators independently extracted the necessary information from the included articles and cross-checked this information. Discrepancies were settled through a discussion with a third investigator (Fangming Zhong).

The following information was collected from each study unless it was unavailable: first author, year of publication, number of patients, patient characteristics (including sex and age), and outcomes (including median PFS, OS, ORR, and DCR; hazard ratios (HRs) for PFS and OS and their 95% confidence intervals (CIs) and AEs such as hypertension and thyroid-stimulating hormone elevation).

#### Risk of bias

The risk of bias was evaluated for each study using Cochrane collaboration’s tool [20]. The same two investigators independently assessed the risk of bias of the included articles. The risk of bias comprised selection bias, performance bias, detection bias, attrition bias, reporting bias, and other biases. When more than 10 studies were included, funnel plots were used to assess the presence of publication bias across the included studies [21].

#### Statistical analysis

RevMan 5.3 software (Copenhagen: The Nordic Cochrane Centre, The Cochrane Collaboration, 2014) was used to perform statistical analysis. The Engauge Digitizer 4.1 software was used to extract survival data from the Kaplan-Meier curves. We calculated pooled HRs for PFS and OS, risk ratios (RRs) for ORRs and DCRs, and odds ratios (ORs) for different AEs. The statistical heterogeneity between trials was evaluated using Q-statistic [22], with a Q-statistic *P* value of <0.1 or an I^2^ of >50% considered to indicate statistically significant between-study heterogeneity [23]. In case of significant heterogeneity, data were analyzed using a random-effects model; otherwise, a fixed-effects model was used. Subgroup analyses were performed on various parameters, such as age, sex, smoking history, disease stage, number of lines of therapy, histology, number of metastases, and EGFR mutation status, to reduce heterogeneity. A sensitivity analysis was performed by removing one article at a time and reporting the analysis results with and without this article. A *P* value of <0.05 was considered statistically significant. An HR of >1 indicated a greater rate of death or progression following treatment with anlotinib, an RR of >1 indicated a greater overall response, and an OR of >1 indicated greater toxicity of therapy. All *P* values were two-sided, and all CIs had two-sided probability coverage of 95%.

## Results

### Characteristics of the studies

**Fig 1:**
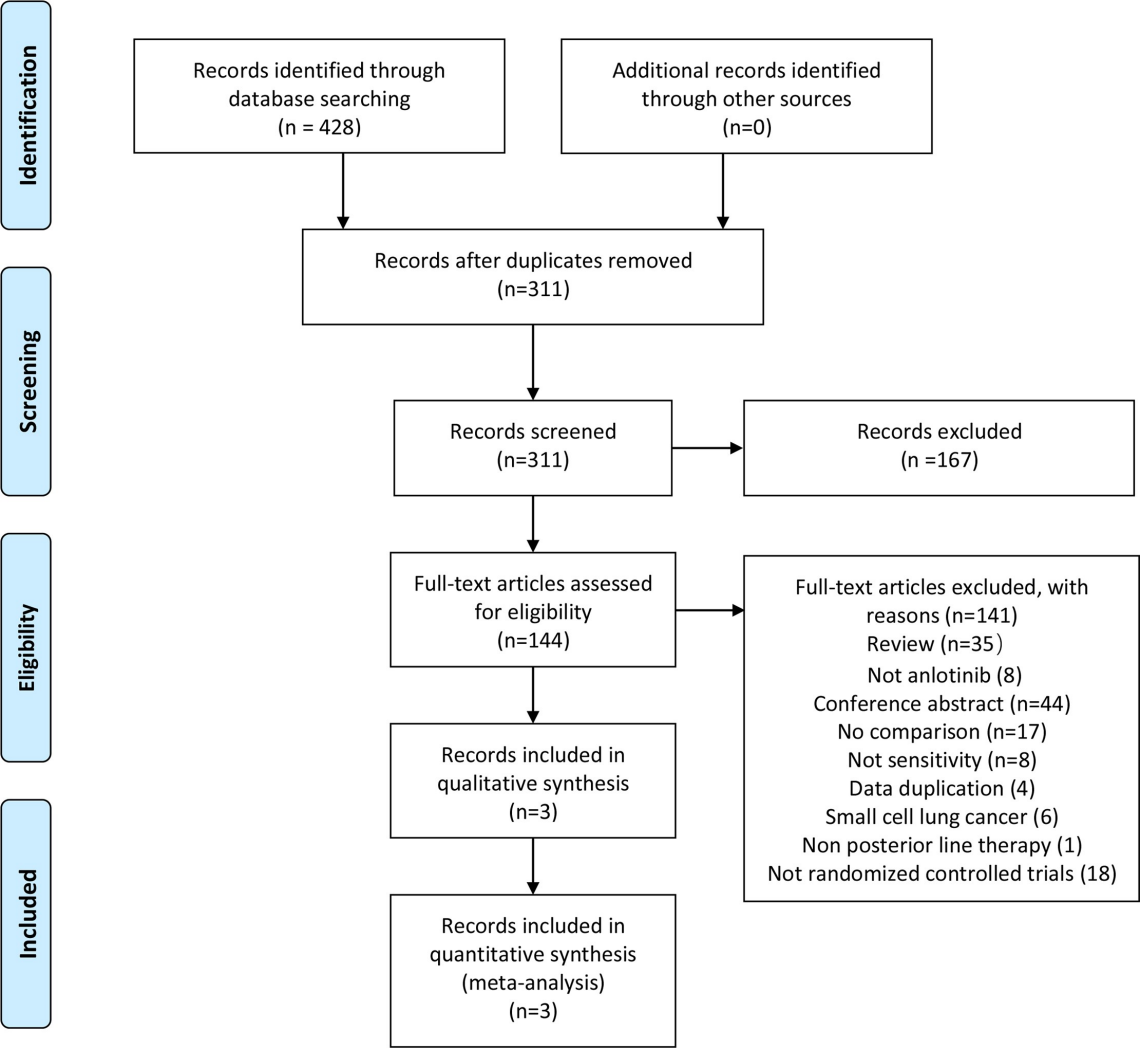
Diagram of the literature search and trial selection process in the meta-analysis. 57, 128, 49, 97, and 97 articles were found in PubMed, the Cochrane Library, Embase, CNKI, and Wanfang database, respectively.

Our search strategy identified 428 candidate articles from relevant databases, and after evaluation of 141 full-text articles, 3 qualified articles describing studies were included based on the inclusion criteria (Fig 1) [24–26]. Three studies were excluded because they presented single-center data from the ALTER 0303 study [27–29]. Two studies were excluded because they were retrospective studies [30,31]. All studies were conducted in China. One article was published in Chinese and the other two in English. In the three qualifying studies, the observation group received oral anirutinib with an identical regimen of 12 mg per day; days 1–14; 21 days per cycle, the control group received placebo treatment. The characteristics of the included studies are shown in Table 1. In total, the included studies comprised 594 patients, and the number of patients per study was 40 to 437. The anlotinib treatment group included a total of 374 patients, 228 of whom were male. The number of patients in each study ranged from 20–294. The placebo treatment group included a total of 220 patients, 142 of whom were male. The number of patients in each study ranged from 20–143.

**Table 1:** The characteristics such as comparison arms, median PFS, median OS, ORR, and DCR of the included studies.

Study	Year	Comparison arms	Participants(male)	Median PFS (mo)	Median OS (mo)	ORR (%)	DCR (%)
Han, B ALTER 0302	2018	Anlotinib	60 (26)	4.8	9.3	10.0	83.3
		Placebo	57 (33)	1.2	6.3	0.0	31.6
Han, B ALTER 0303	2018	Anlotinib	294(188)	5.4	9.6	9.2	81.0
		Placebo	143(97)	1.4	6.3	0.7	37.1
Dai X	2019	Anlotinib	20(14)	5.0	8.0	15.0	85.0
		Placebo	20(12)	2.0	6.0	0.0	30.0

PFS: Progression-free survival, OS: Overall survival, ORR: Objective response rate, DCR: Disease control rate.

### Risk of bias

**Fig 2:**
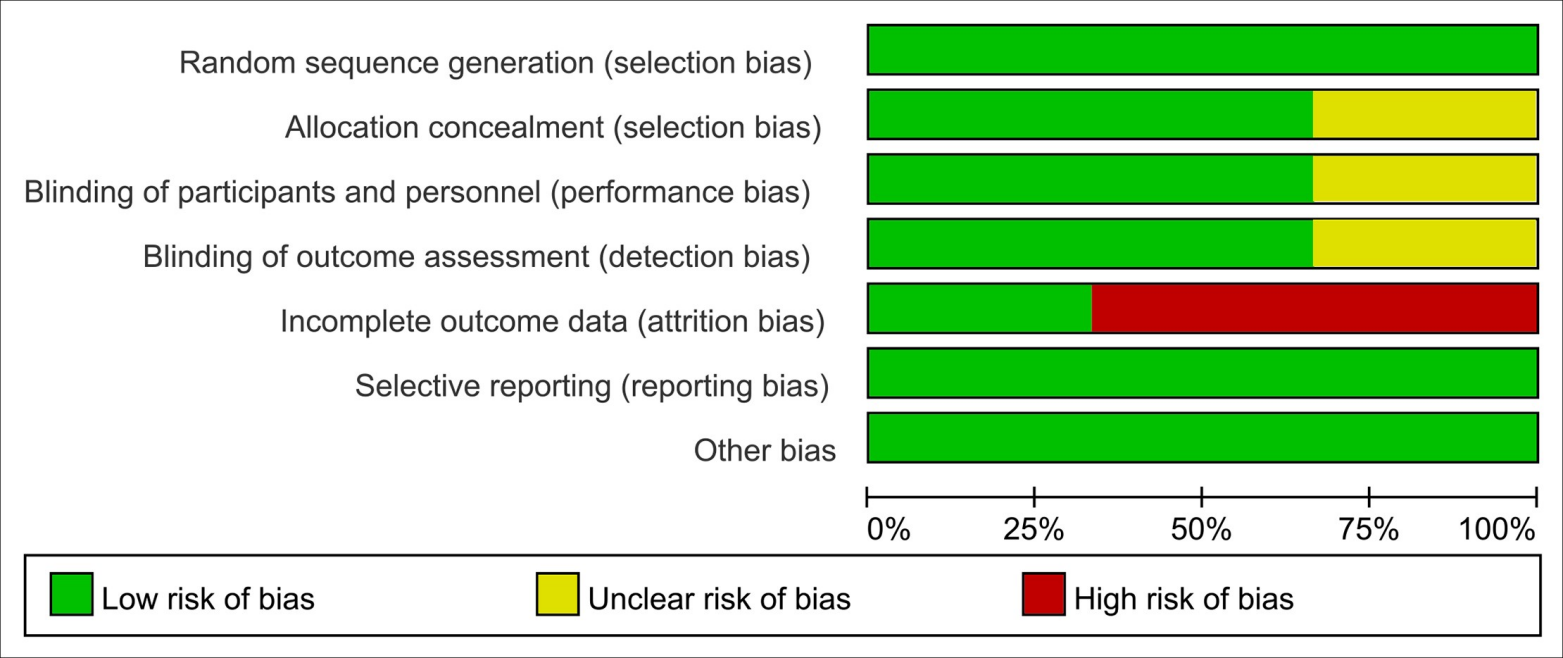
Diagram of the risk of bias in the included studies.

All of the RCTs had a low risk of bias for randomization. Most RCTs had a low risk of bias for allocation concealment (67%), and 33% of the studies did not report details on allocation concealment. The risk of bias due to blinding of participants and personnel and blinding of the outcome assessment were unclear for 33% of studies. Additionally, 67% of studies did not completely report partial outcome data; these RCTs had a high risk of bias for incomplete outcome data. Results of the analyses of risk of bias in these studies were illustrated in Fig 2. The summary of the risk of bias for each study is shown in S1 Fig. Less than 10 studies were included; therefore, publication bias was not assessed across the included studies.

### Progression-free survival

**Fig 3:**
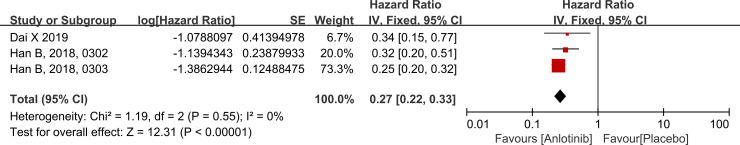
Forest plot comparing the PFS between anlotinib and placebo group in advanced NSCLC. The red squares represent the HR of a study, and the black line their confidence intervals. The black diamond represent the pooled HR and its confidence interval.

All of the three RCTs evaluated PFS, as time to progression. The fixed-effects model used to estimate the pooled HR for PFS revealed that anlotinib administration significantly improved PFS in patients with advanced NSCLC compared with placebo (HR: 0.27, 95% CI: 0.22–0.33, *P* < 0.001; Fig 3). There was no significant heterogeneity in PFS rate between the two studies (*P* = 0.55, I^2^ = 0%). We also performed subgroup analysis based on the subgroup of age, sex, smoking history, disease stage, number of lines of therapy, histology, number of metastases, and EGFR mutation status to observe whether different disease states have an effect on the efficacy of anlotinib. The results of subgroup analysis for each subgroup were presented in S2 Fig. Improvements in PFS following anlotinib administration were observed in all subgroups (*P* < 0.05).

### Overall survival

**Fig 4:**
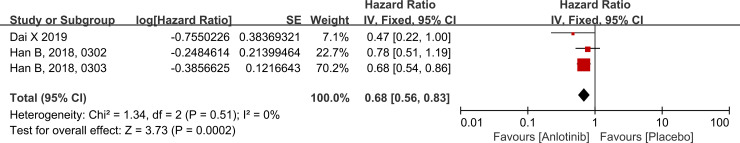
Forest plot comparing the OS between the anlotinib and placebo groups with advanced NSCLC. The red squares represent the HR of a study, and the black line their confidence intervals. The black diamond represent the pooled HR and its confidence interval.

Three RCTs reported OS. The pooled HR for OS calculated using the fixed-effects model showed that anlotinib administration significantly improved OS in patients with advanced NSCLC compared with placebo (HR: 0.68, 95% CI: 0.56–0.83, *P* < 0.001; Fig 4). There was no significant heterogeneity in OS between the included studies (*P* = 0.51, I^2^ = 0%). Only one study reported OS data for each subgroup, so a meta-analysis could not be performed.

### Objective response rate

**Fig 5:**
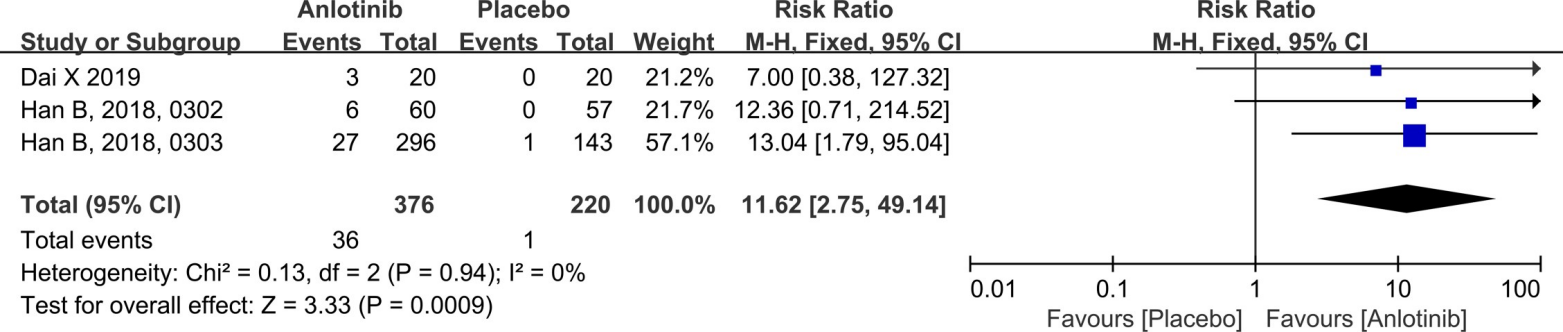
Forest plot comparing the ORR between anlotinib and placebo group in advanced NSCLC. The blue squares represent the RR of a study, and the black line their confidence intervals. The black diamond represent the pooled RR and its confidence interval.

Three RCTs assessed ORR. The pooled RR for ORR calculated using the fixed-effects model was 11.62 and was significantly higher in the anlotinib group than in the placebo group (95% CI: 2.75–49.14, *P* < 0.001; Fig 5), indicating that anlotinib administration in patients with advanced NSCLC improved ORR. There was no significant difference in ORR between the studies (*P* = 0.94, I^2^ = 0%). All studies did not report subgroup ORRs, so subgroup analysis was not performed.

### Disease control rate

**Fig 6:**
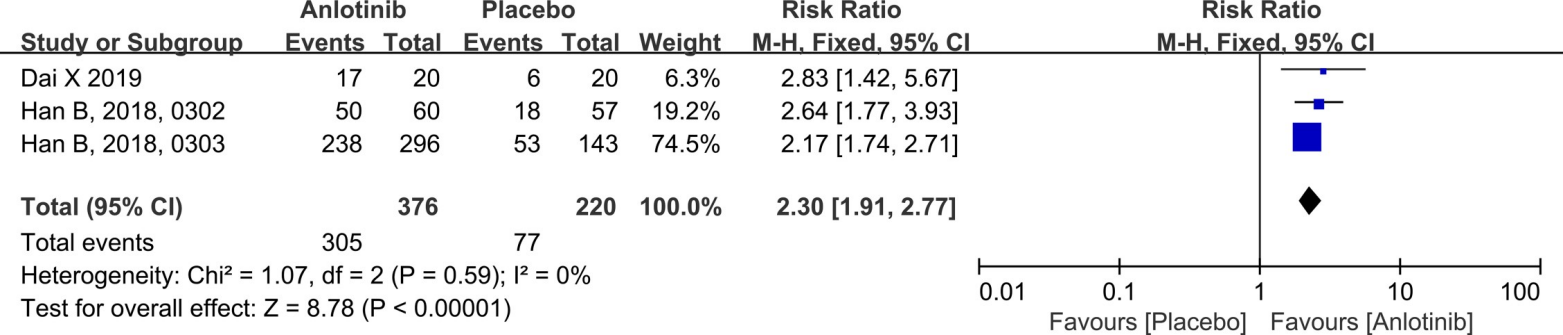
Forest plot comparing the DCR between the anlotinib and placebo groups with advanced NSCLC. The blue squares represent the RR of a study, and the black line their confidence intervals. The black diamond represent the pooled RR and its confidence interval.

Three RCTs assessed DCR. The total pooled RR for DCR calculated using the fixed-effects model was 2.30 and was significantly higher in patients with NSCLC who received anlotinib than in those who received placebo (95% CI: 1.91–2.77, *P* < 0.001; Fig 6). The DCR did not differ significantly between the studies (*P* = 0.59, I^2^ = 0%). No studies reported subgroup DCRs. Therefore, a subgroup analysis could not be performed.

### Sensitivity analysis

We observed that one study (Han B 2018 303) comprised the major part of the analysis by contributing more than 60% of the weight of the pooled analysis, which might have caused the results to be significant. Therefore, we conducted a sensitivity analysis, which indicated that the removal of any one of the three included articles did not affect the final results.

### Toxicity

Two included articles reported the incidence of AEs in detail, one article did not report the incidence of AEs in the placebo group. Hypertension, fatigue, thyroid-stimulating hormone (TSH) elevation, anorexia, hyper triglyceridemia, hand-foot syndrome (HFS), hyper cholesterolemia, diarrhea, and hemoptysis were the most common AEs with statistical difference between the anlotinib and placebo groups. Heterogeneity between the groups was insignificant for each AE except fatigue; therefore, a random-effects model was employed for fatigue and a fixed-effects model for all other AEs. For fatigue, the pooled OR was 1.83 (95% CI: 0.76–4.40; S3 Fig), indicating that fatigue was lower in the placebo group than in the anlotinib group, but this difference was not statistically significant (*P* = 0.18). Significant heterogeneity (I^2^ = 75%) was detected between the anlotinib and placebo groups. For hypertension, TSH elevation, anorexia, hypertriglyceridemia, HFS, hypercholesterolemia, diarrhea, and hemoptysis, the pooled ORs were 12.01 (95% CI: 7.55–19.09), 11.13 (95% CI: 6.11–20.29), 1.64 (95% CI: 1.11–2.42), 2.90 (95% CI: 1.90–4.42), 8.82 (95% CI: 4.91–15.84), 4.63 (95% CI: 2.84–7.53), 3.44 (95% CI: 2.12–5.58), and 2.89 (95% CI: 1.60–5.19), respectively (S4 Fig), indicating less hypertension, TSH elevation, anorexia, hypertriglyceridemia, HFSR, hypercholesterolemia, diarrhea, and hemoptysis in the placebo group compared with the anlotinib group. Additionally, the differences were statistically significant for all AEs (*P* < 0.05). Heterogeneity (I^2^ < 50%) was insignificant between the anlotinib and placebo groups. For AEs classified as grade 3 or higher, the pooled OR was 2.94 (95% CI: 1.99–4.35; S5 Fig), indicating that AEs of grade 3 or higher were more prevalent in the anlotinib group than in the placebo group and the difference was statistically significant (*P* < 0.001). Heterogeneity (I^2^ = 0%) was insignificant between the anlotinib and placebo groups.

## Discussion

In recent years, the use of targeted therapies and immunotherapy has considerably prolonged the survival period of patients with advanced NSCLC [32,33]. However, the prognosis remains poor for patients without targeted gene mutations after systemic treatment or who exhibit progression after targeted therapy and immunotherapy. Presently, there is no standard treatment for patients with advanced NSCLC who suffer from disease progression after at least two lines of systemic therapy [9]; thus, the medical needs of these patients are not met. Anti-angiogenesis is an important therapeutic strategy for solid tumors [34]. Apatinib is an oral small-molecule VEGFR inhibitor, which offers substantial clinical benefits for patients with advanced NSCLC [14]. However, this drug has not been approved by the China National Medical Products Administration (NMPA).

Compared with apatinib, anlotinib, a multiple-target TKI developed by the Chia Tai Tianqing Pharmaceutical Group Co., Ltd. (Lianyungang, Jiangsu, China), targets VEGFR1–3, FGFR1–4, PDGFR α/β, and c-Kit among others. The multiple targets of anlotinib other than angiogenic kinases might contribute to its increased anti-cancer efficacy. Several studies have reported the utility of anlotinib therapy for patients with advanced NSCLC [18,24,25], and the drug has been approved by the China NMPA as a third-line therapy for advanced NSCLC. Until now, most studies on the efficacy of anlotinib for advanced NSCLC have been conducted in China; therefore, all the papers evaluated in the present study were authored in China. To the best of our knowledge, the present study is the first meta-analysis to assess the clinical benefit and AEs of anlotinib as a third- or later line treatment in patients with advanced NSCLC. Because there is no standard third- or later line treatment, we included currently available studies that utilized a placebo as the control compared with anlotinib as a post-second-line treatment for advanced NSCLC. Our results confirm that anlotinib provides substantial clinical benefits for patients with advanced NSCLC in terms of PFS, OS, ORR, and DCR; the pooled HRs for PFS and OS indicated that anlotinib administration can effectively prolong survival in such patients. The heterogeneity between studies in terms of PFS, OS, ORR, and DCR was insignificant. Moreover, subgroup analysis showed that PFS improves following anlotinib therapy, regardless of age, sex, smoking history, disease stage, number of therapy line, histology, number of metastases, or EGFR mutation status. In the ALTER 0302 trial, positive results were not observed in terms of OS, and subgroup data were not reported. Therefore, no meta-analysis was performed on the subgroup data. The ALTER 0303 trial showed that improvement in OS due to anlotinib occurs across most predefined subgroups (such as age, number of therapy line, number of metastases, and EGFR mutation status). In the present study, ORR and DCR were significantly higher in the anlotinib group than in the placebo group. No study reported subgroup ORRs and DCRs; therefore, subgroup analysis was not performed. Furthermore, the improvements in PFS, OS, ORR, and DCR are meaningful for patients with advanced NSCLC; therefore, approval of anlotinib by the China NMPA as a third- or later line treatment for patients with advanced NSCLC is reasonable. The present meta-analysis demonstrates that clinical benefits may be observed in such patients, regardless of age, histology, number of metastases, or EGFR mutation status. Thus, anlotinib might represent a new option for the treatment of patients with advanced NSCLC when first- and second-line treatments are unsuccessful.

The incidence of AEs (including those of grade 3 or above) except fatigue was higher in the anlotinib group than in the placebo group, but heterogeneity was insignificant among the other subgroups (hypertension, TSH elevation, anorexia, hypertriglyceridemia, HFSR, hypercholesterolemia, diarrhea, and hemoptysis). This might be related to the use of placebo in the control group, AEs related to the drug still need to be treated with caution, especially some of the intolerable grade 3 or above AEs. However, on the whole, AEs associated with anlotinib were controllable and the advantages of the use of anlotinib for advanced NSCLC outweigh the disadvantages.

There were several limitations in our study. First, our meta-analysis was based on data extracted from published literature, rather than individual patient data. Second, the number of included studies was low; more high-quality RCTs are warranted to assess the efficacy and AEs of anlotinib for the treatment of patients with advanced NSCLC. In addition, all the included studies were done on only one country as the treatment might fail in another country with different ethnicity and socioeconomic levels.

In summary, anlotinib is an effective choice for the treatment of patients with refractory advanced NSCLC and provides clinical benefits in terms of improvements in PFS, OS, ORR, and DCR. AEs identified to be associated with anlotinib were tolerable. Therefore, treatment with anlotinib can represent a third- or later line treatment for such patients.

## Supporting information

S1 Checklist PRISMA checklist. (DOC)

S1 Fig Risk of bias summary. (TIF)

S2 Fig Forest plot of PFS subgroups. (TIF)

S3 Fig Forest plot of fatigue. (TIF)

S4 Fig Forest plot of other toxicity. (TIF)

S5 Fig Forest plot of toxicity of grade 3 or higher. (TIF)

S1 File Search strategy. (DOCX)

S2 File Funding. (DOCX)
