# Supplementary figures and images for: Anlotinib for refractory advanced non-small-cell lung cancer: A systematic review and meta-analysis
Source: PLoS One. 2020 Nov 30;15(11):e0242982. doi: 10.1371/journal.pone.0242982 (PMC7703897; doi:10.1371/journal.pone.0242982)

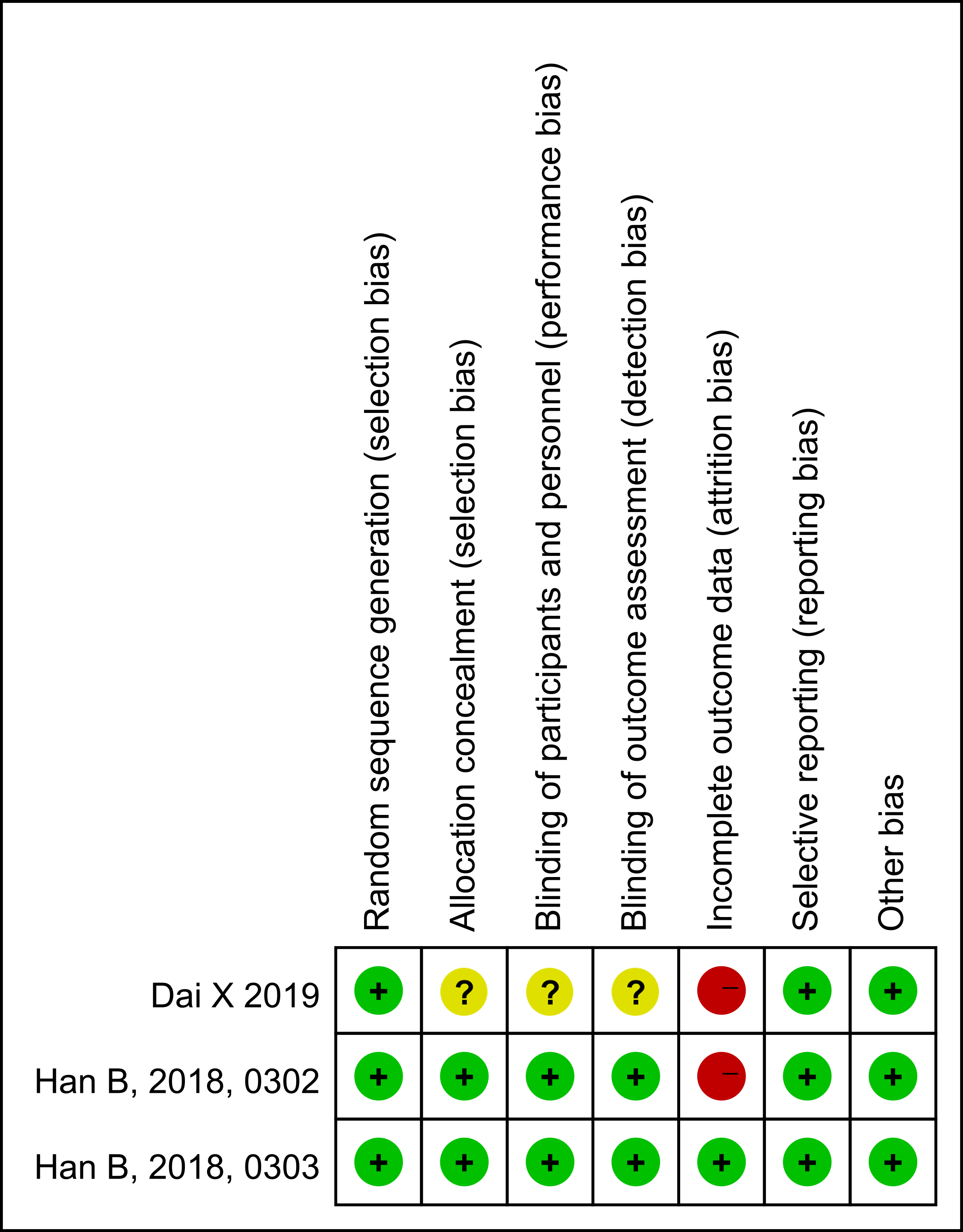

Supplement: S1 Fig — (TIF) [file pone.0242982.s002.tif]

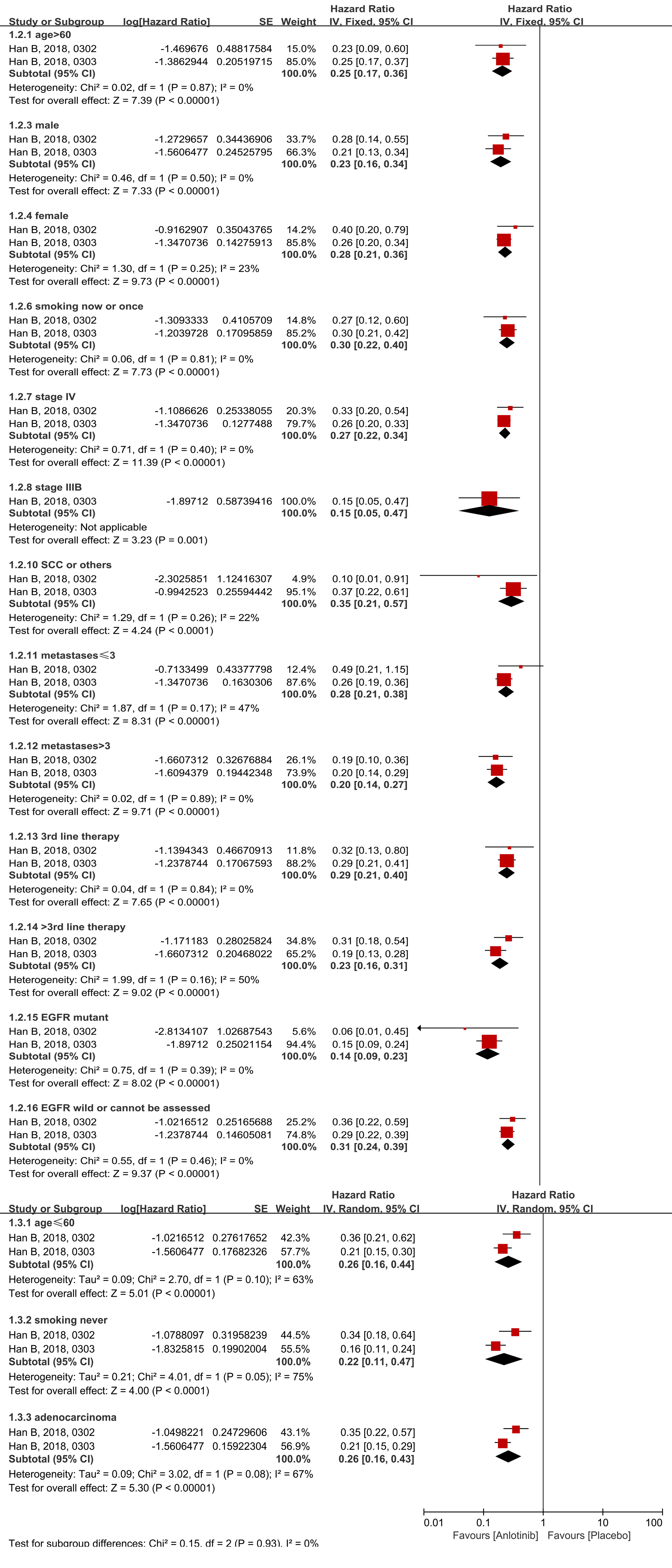

Supplement: S2 Fig — (TIF) [file pone.0242982.s003.tif]

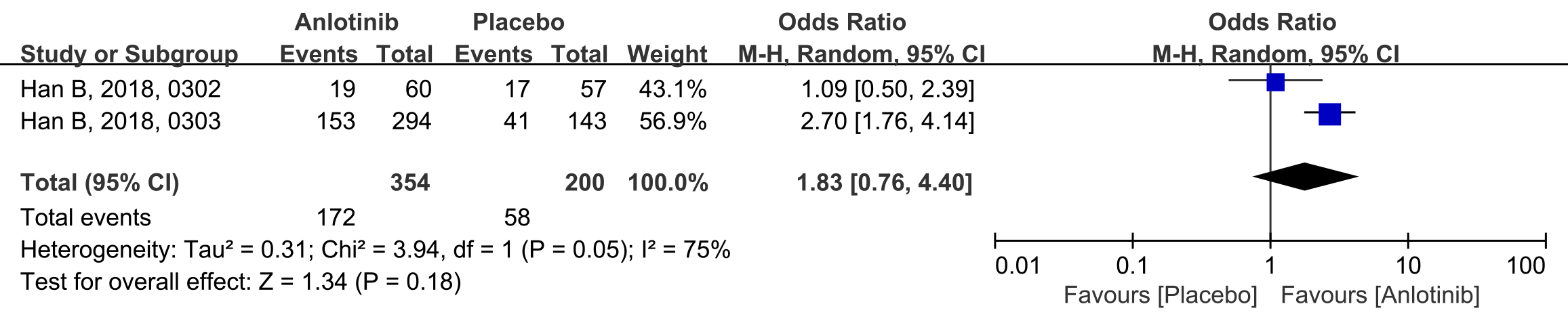

Supplement: S3 Fig — (TIF) [file pone.0242982.s004.tif]

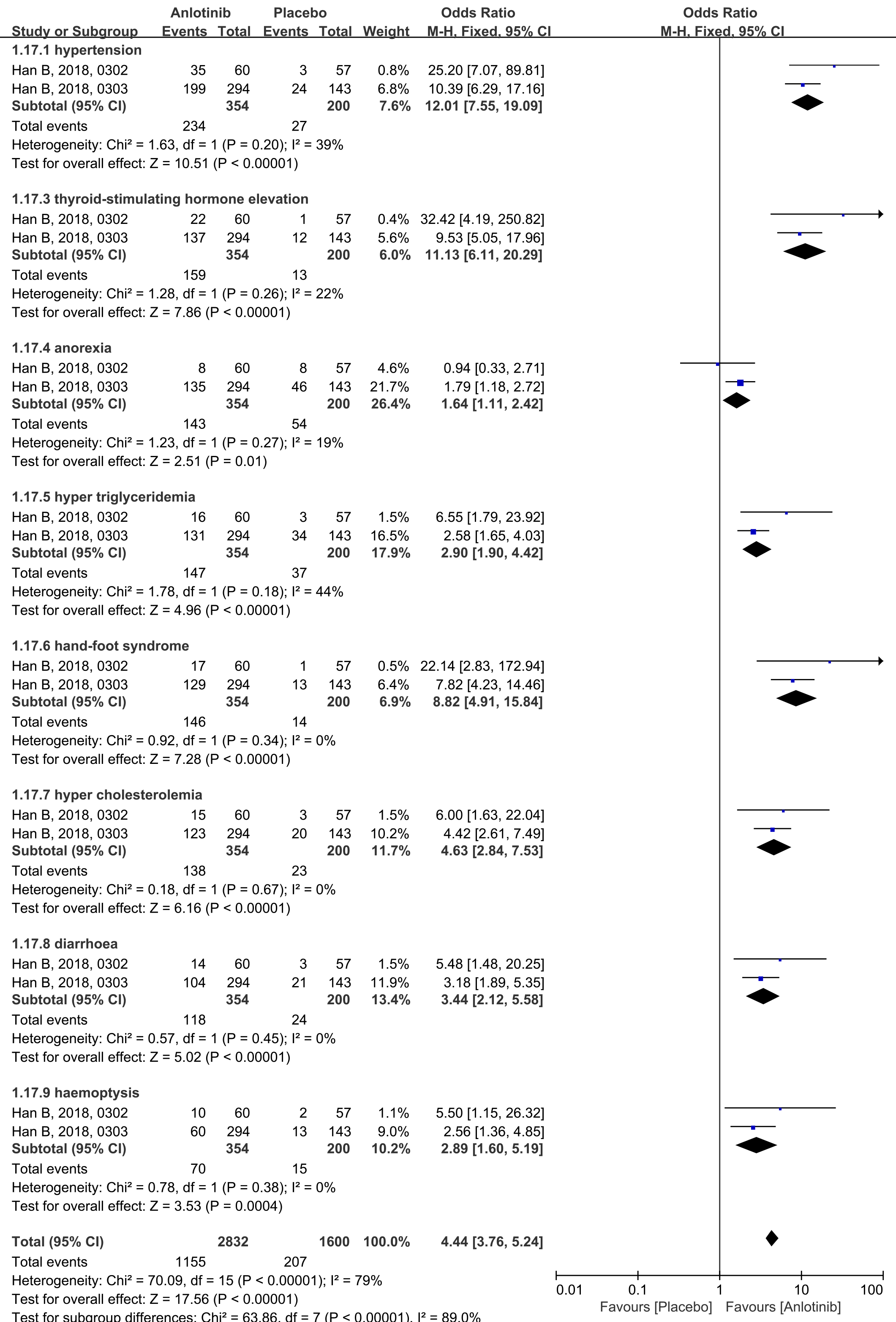

Supplement: S4 Fig — (TIF) [file pone.0242982.s005.tif]

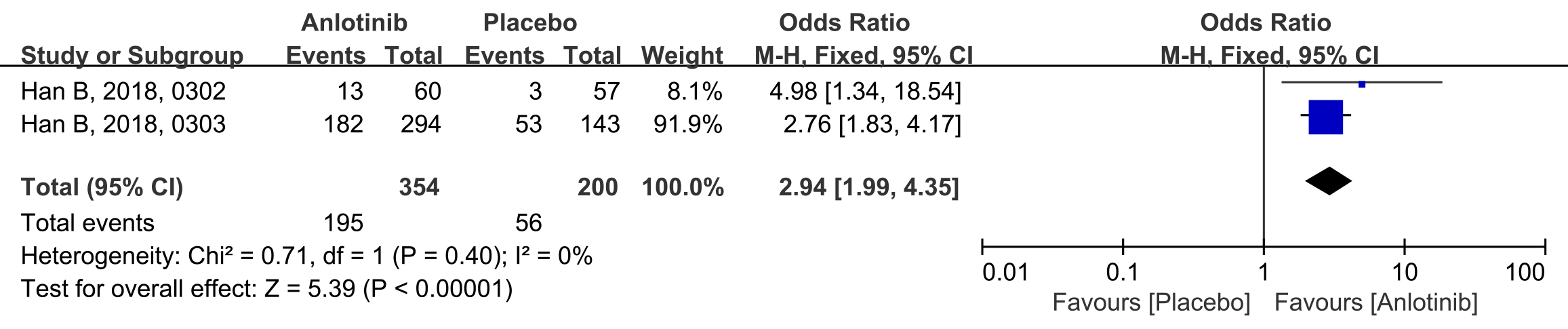

Supplement: S5 Fig — (TIF) [file pone.0242982.s006.tif]

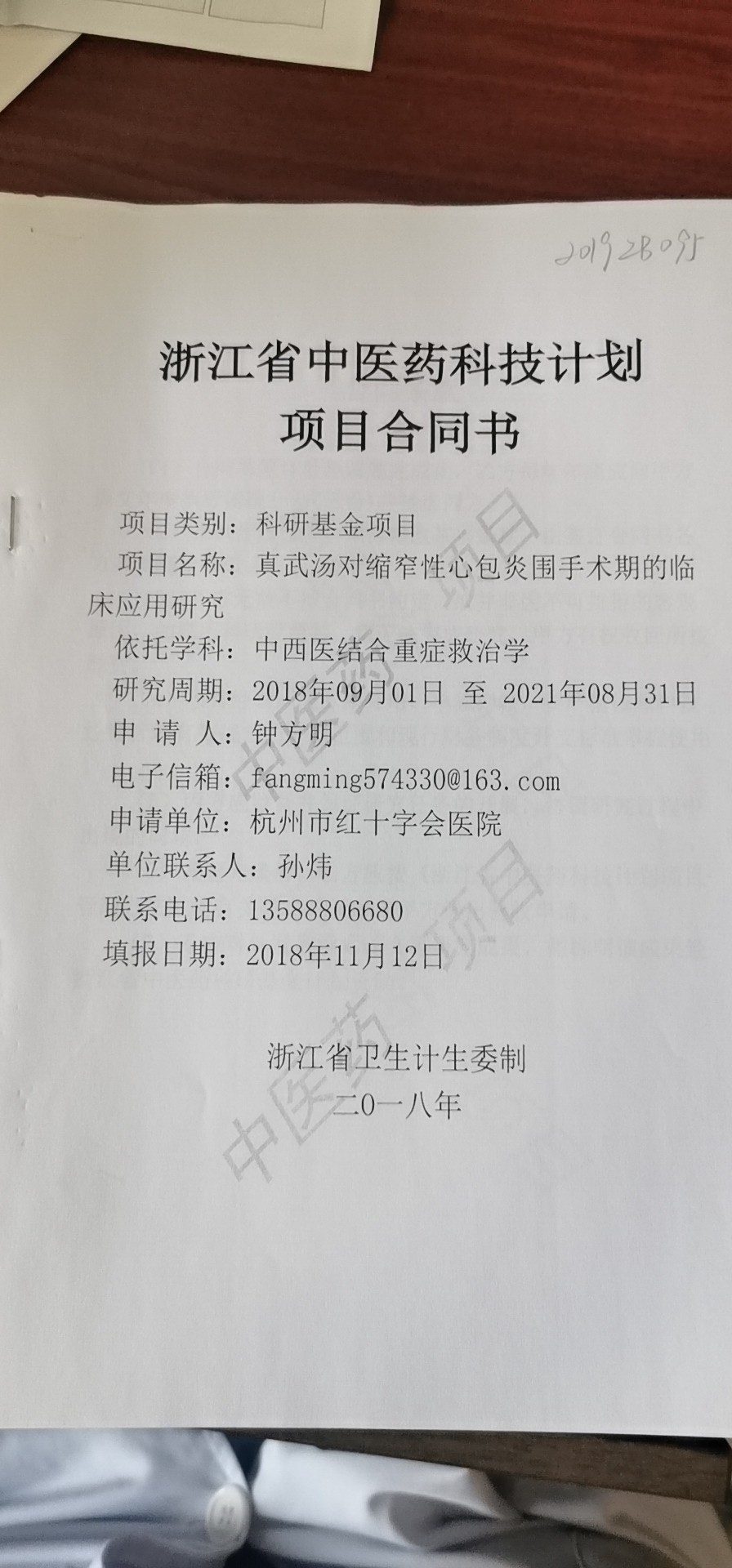


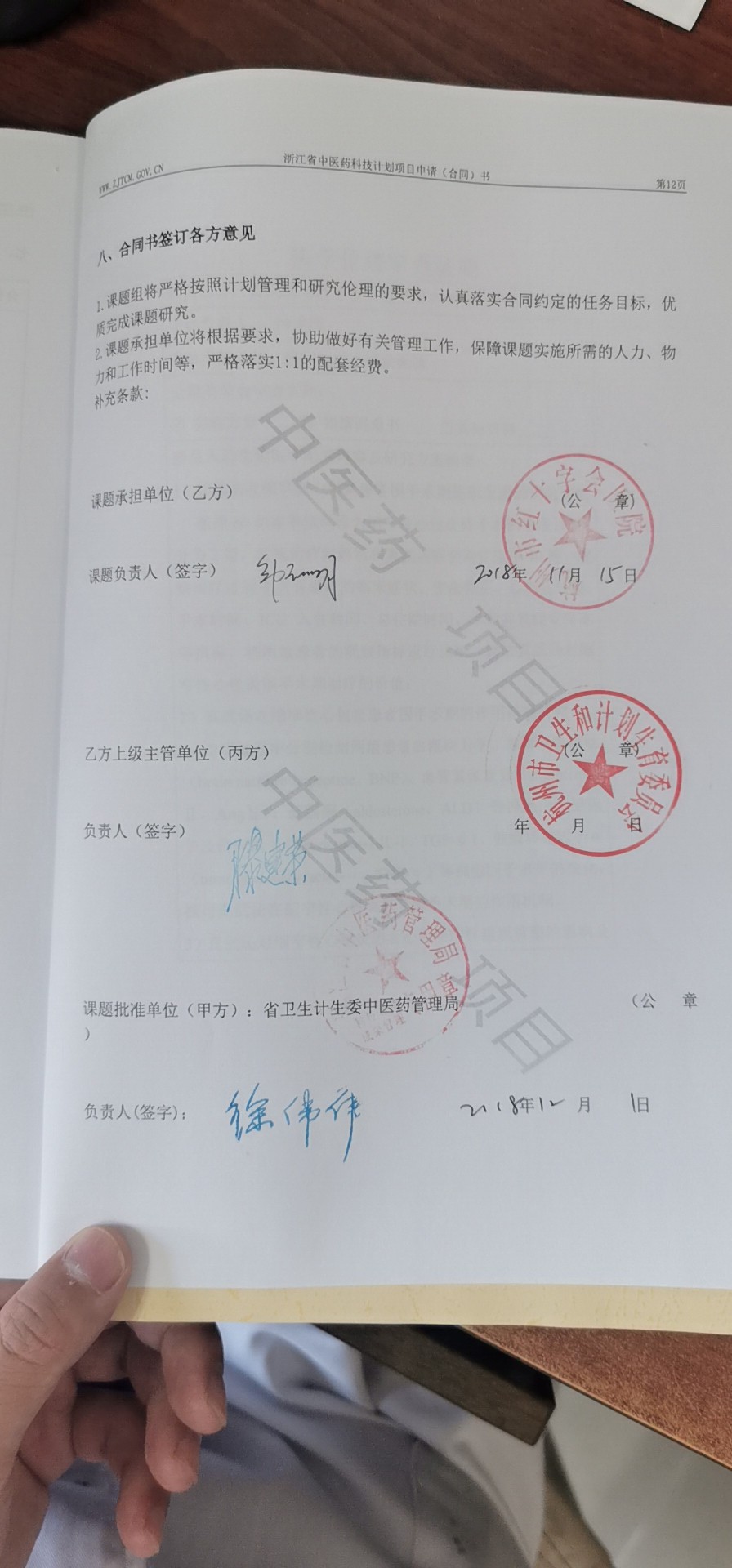

Supplement: S2 File — (DOCX) [file pone.0242982.s008.docx]
