# Supplementary material for: Anlotinib for refractory advanced non-small-cell lung cancer: A systematic review and meta-analysis
Source: PLoS One. 2020 Nov 30;15(11):e0242982. doi: 10.1371/journal.pone.0242982 (PMC7703897; doi:10.1371/journal.pone.0242982)
Supplement: S1 File — (DOCX) [file pone.0242982.s007.docx]

Pubmed and Cochrane:

#1 "Carcinoma, Non-Small-Cell Lung"[Mesh]

#2 “Carcinoma, Non Small Cell Lung” OR “Carcinomas, Non-Small-Cell Lung” OR “Lung Carcinoma, Non-Small-Cell” OR “Lung Carcinomas, Non-Small-Cell” OR “Non-Small-Cell Lung Carcinomas” OR “Nonsmall Cell Lung Cancer” OR “Non-Small-Cell Lung Carcinoma” OR “Non Small Cell Lung Carcinoma” OR “Carcinoma, Non-Small Cell Lung” OR “Non-Small Cell Lung Cancer”

#3 #1 OR #2

#4 "anlotinib" [Supplementary Concept] OR AL3818

#5 #3 AND#4

Embase

#1 'non small cell lung cancer'/exp

#2 ‘Carcinoma, Non Small Cell Lung’ OR ‘Carcinomas, Non-Small-Cell Lung’ OR ‘Lung Carcinoma, Non-Small-Cell’ OR ‘Lung Carcinomas, Non-Small-Cell’ OR ‘Non-Small-Cell Lung Carcinomas’ OR ‘Nonsmall Cell Lung Cancer’ OR ‘Non-Small-Cell Lung Carcinoma’ OR ‘Non Small Cell Lung Carcinoma’ OR ‘Carcinoma, Non-Small Cell Lung’ OR ‘Non-Small Cell Lung Cancer’

#3 #1 OR #2

#4 'anlotinib'/exp OR AL3818

#5 #3 AND #4

CNKI and Wanfang

#1 非小细胞肺癌 OR 肺恶性肿瘤

#2 安罗替尼 OR AL3818

#3 #1 AND #2
